# Chrysin Induced Cell Apoptosis Through *H19*/let-7a/*COPB2* Axis in Gastric Cancer Cells and Inhibited Tumor Growth

**DOI:** 10.3389/fonc.2021.651644

**Published:** 2021-06-03

**Authors:** Lin Chen, Qirong Li, Ziping Jiang, Chengshun Li, Haobo Hu, Tiedong Wang, Yan Gao, Dongxu Wang

**Affiliations:** ^1^ Laboratory Animal Center, College of Animal Science, Jilin University, Changchun, China; ^2^ Department of Hand Surgery, The First Hospital of Jilin University, Changchun, China

**Keywords:** *H19*, let-7a, *COPB2*, chrysin, gastric cancer

## Abstract

**Background:**

Chrysin is a natural flavone that is present in honey and has exhibited anti-tumor properties. It has been widely studied as a therapeutic agent for the treatment of various types of cancers. The objectives of this present study were to elucidate how chrysin regulates non-coding RNA expression to exert anti-tumor effects in gastric cancer cells.

**Methods:**

Through the use of RNA sequencing, we investigated the differential expression of mRNAs in gastric cancer cells treated with chrysin. Furthermore, *COPB2, H19* and let-7a overexpression and knockdown were conducted. Other features, including cell growth, apoptosis, migration and invasion, were also analyzed. Knockout of the *COPB2* gene was generated using the CRISPR/Cas9 system for tumor growth analysis *in vivo*.

**Results:**

Our results identified *COPB2* as a differentially expressed mRNA that is down-regulated following treatment with chrysin. Moreover, the results showed that chrysin can induce cellular apoptosis and inhibit cell migration and invasion. To further determine the underlying mechanism of COPB2 expression, we investigated the expression of the long non-coding RNA (lncRNA) *H19* and microRNA let-7a. Our results showed that treatment with chrysin significantly increased let-7a expression and reduced the expression of *H19* and *COPB2*. In addition, our results demonstrated that reduced expression of *COPB2* markedly promotes cell apoptosis. Finally, *in vivo* data suggested that *COPB2* expression is related to tumor growth.

**Conclusions:**

This study suggests that chrysin exhibited anti-tumor effects through a *H19*/let-7a/*COPB2* axis.

## Introduction

Currently, gastric cancer (GC) is the third most common cause of human mortality among malignant cancers (1). Although surgical treatment for GC has led to increased survival rates, the diagnosis of GC needs to improve (2). There are several potential biomarkers of GC, including many genes and cell signaling pathways that are involved in GC development, such as *BRCA2* and Ras-Raf-MAPK signaling (3, 4). Coatomer protein complex subunit beta 2 (COPB2) is a protein that functions to transport other proteins as vesicles from the endoplasmic reticulum to Golgi apparatus (5). Recently, numerous reports have indicated that *COPB2* is abnormally expressed in colorectal cancer (CRC), cholangiocellular carcinoma and lung cancer (6–8). Previous studies have indicated that the reduction of *COPB2* expression inhibited cell growth and induced apoptosis through the JNK/c-Jun signaling pathway in RKO and HCT116 cells (9). Moreover, miR4461 and miR335 were found to regulate *COPB2* expression, which subsequently inhibited cell growth in CRC and lung cancer cells (10, 11).

Increasing evidence suggests that non-coding RNAs (ncRNAs), such as miRNAs, can be applied to the classification of GC (12). As tumor suppressors, the let-7 family is down-regulated in GC (13). Increased expression of let-7a has been shown to inhibit cell migration and invasion in prostate cancer (14). Compared to the loss of let-7a expression, the long non-coding RNA (lncRNA) *H19* has been shown to be highly expressed in cancers, including GC (15). As a molecular sponge, *H19* was found to be related to let-7 in the context of breast cancer stem cells (16). Previous reports have suggested that *H19* expression suppressed endogenous let-7 while *H19* mutant was not related to let-7 (17). Additionally, reduced expression of *H19* induced cellular apoptosis and inhibited cell growth in HCC (18). However, there is little evidence to suggest that *COPB2* expression is associated with lncRNAs and miRNAs in GC.

As a traditional Chinese medicine, chrysin is a natural flavone that has anti-cancer function (19). Previous reports have indicated that chrysin induces cellular apoptosis and inhibits tumor glycolysis in HCC (20). Moreover, chrysin has been shown to inhibit cell migration and invasion in melanoma cells (21). In this study, chrysin was used to treat GC cells and we screened differentially expressed genes using RNA-seq. Additionally, we created a *COPB2* knockout (KO) cell line using the CRISPR/Cas9 system. Our findings indicate that chrysin can regulate *COPB2* expression through let-7, which is antagonized by *H19*.

## Materials and Methods

### Cell Culture and Chrysin Treatment

Human GC cell lines (SGC7901, MKN45 and BGC823) and the human gastric epithelial cell line GES-1 were grown in Dulbecco’s modified Eagle’s (DMEM; Gibco) supplemented with 10% fetal bovine serum (Gibco), and cultured at 37°C in 5% CO_2_. The human gastric epithelial cell GES1 served as control. Experiments were conducted by treating GES1, SGC7901, MKN45 and BGC823 cells with 40 μM of Chrysin (Yuanye Bio-Technology, Shanghai) for 48 h when they reached 80 to 90% confluence.

### RNA Isolation and RNA-Seq Analysis

GC cells were treated with 40 μM chrysin for 48 h, after which total RNA was extracted. To remove and purify ribosomal RNA (rRNA), we used the RiboZero Magnetic Gold Kit (Epidemiology, USA). Then, RNA-seq (Sangon Biotech, Shanghai, China) was carried out on HiSeq2500 (Illumina, USA) to analyze raw reads, which were quality controlled by FastQC. Using the HISAT2 software, the sequenced-reads were aligned to the reference sequence. The gene expression analysis and differential gene expression analysis were determined using DEGseq and DESeq program, respectively, in HISAT2 (qValue <0.05, Fold Change >2). Using clusterProfiler, the enrichment analysis, including Kyoto Encyclopedia of Genes and Genomes (KEGG) pathway, of differential expressed genes was determined.

### Knockdown and Overexpression of *COPB2, H19* and let-7a

The pcDNA3.1 (GenePharma, China) vector served as the backbone for the overexpression construct of *COPB2* (pcDNA3.1-COPB2) and *H19* (pcDNA3.1-H19). The cells were cultured without FBS once they reached a confluence of 80% over 12–16 h. Next, the pcDNA3.1-COPB2 (2 μg), pcDNA3.1-H19 (2 μg) and Lipofectamine 2000 (Invitrogen, USA) were utilized for transfection. After incubating for 48 h, G418 (400 mg/ml, Invitrogen, USA) was added to GC cells. The clones were grown and picked after 14 days.

The siRNAs of *COPB2* (si-COPB2) and *H19* (si-H19) were obtained from RiboBio (Guangzhou, China). The target sequences of small interfering RNAs (siRNAs) are listed in **Table S1**. The mimics and inhibitors of let-7a-3p, miR29b-3p and miR675-3p were obtained from RiboBio. The GC cells were transfected with knockdown (siRNA), let-7a-3p mimics, and let-7a-3p inhibitor for 48 h. The nonspecific siRNA (si-Nc) was transfected into control cells.

The COPB2-KO was generated using the CRISPR/Cas9 system (px459, Addgene, USA). The single guide RNAs (sgRNAs) were designed as previously reported (22). The sequences of sgRNAs are listed in **Table S2**. Transfection was conducting using COPB2-KO (5 μg) and Lipofectamine 2000 (Invitrogen, USA) for 48 h. Next, puromycin helped select the positive clones. After 14 days, the clones (COPB2-KO) were grown and picked for subsequent western blot, qPCR and sequencing analysis.

### DNA Methylation Analysis

The Bisulfite Sequencing PCR (BSP) protocol was carried out as previously described (23). Using the TIANamp Genomic DNA Kit (TIANGEN, Beijing, China), the DNA of SGC7901 and BGC823 cells were extracted. The DNA was modified using CpGenome™ Turbo Bisulfite Modification Kit (Millipore, USA). The differentially methylated regions (DMRs) of *H19* were amplified using nested PCR. The products, which included 10 positive clones, were analyzed using the BiQ Analyzer software (http://biq-analyzer.bioinf.mpi-inf.mpg.de/tools/MethylationDiagrams/index.php). The primers of *H19* DMR are listed in **Table S3**.

### Gene Expression Analysis

The GC cells’ RNA was extracted and cDNAs were generated using the cDNA first-strand synthesis kit (TIANGEN, China). Gene expression analysis was conducted using quantitative real-time PCR (qPCR). The conditions for qPCR included heating to 94°C for 3 min, and then denaturation at 94°C for 10 s after 35 cycles. The annealing was carried out at 59°C for 15 s. The products were extended at 72°C for 30 s. The internal controls included GAPDH and U6 for genes and miRNAs, respectively. The primers for qPCR are listed in **Table S4**. The sequences of *COPB2* exon 5 and exon 22 are listed in **Table S5**.

### Cell Migration and Invasion Analysis

Wound healing assay was conducted to analyze cell migration of GC cells. In brief, 5 × 10^5^ cells were cultured and seeded before treatment with chrysin, siRNAs, overexpression vectors, let-7a-3p mimics or let-7a-3p inhibitor. The cells were cultured after a scratched line was created with culture medium without serum. Cell migration was measured using the scratched area at 12, 24 and 48 h.

For cell invasion assays, GC cells (3 × 10^4^) were cultured and seeded with 20 μl Matrigel prior to treatment with chrysin, siRNAs, overexpression vectors, let-7a-3p mimics and let-7a-inhibitor (BD Biosciences, USA). Next, 0.5 ml of medium, which contained 10% FBS, was added to the cells for 24 h. Then, 0.2% crystal violet dye (Solarbio, China) was used to stain cells after being fixed with 4% paraformaldehyde. The stained cells were assayed using the ImageJ software.

### Cell Counting Kit-8 Assay

The GC cells (4 × 10^3^) were cultured and seeded prior treatment with chrysin, siRNAs, overexpression vectors, let-7a-3p mimics and let-7a-3p inhibitors in order to conduct cell viability assay, as previously described (24). Then, Cell Counting Kit-8 (CCK-8) solution (10 μl, Dojindo, Kumamoto, Japan) was added to each well. After incubating for 2.5 h, the cells were measured using absorbance (OD) at 450 nm to analyze cell viability.

### Cell Apoptosis Analysis

GC cells (1 × 10^6^) were cultured prior to treatment with chrysin, siRNAs, overexpression vectors, let-7a-3p mimics and let-7a-inhibitor for detection of cellular apoptosis, as previously described (25). Then, Annexin V-FITC/PI reagent was added to cell to react for 30 min and flow cytometry (BD Biosciences, Franklin Lakes, NJ, USA) was used to detect fluorescent cells.

### Western Blot Analysis

Total protein was extracted from GC cells using protein extraction buffer (Beyotime, China). Then, proteins were quantified utilizing the BCA protein assay kit (TIANGEN, Beijing, China). Sodium dodecyl sulfate-polyacrylamide gel electrophoresis was used to separate the proteins. After electrophoresis, proteins were transferred to the polyvinylidene difluoride (PVDF) membrane. The membrane was then incubated with primary antibodies, including anti-COPB2 (BETHYL, A304-522A-M-1, USA), anti-P53 (Abcam, ab131442, USA), anti-BAX (CST, D2E11, USA), anti-BCL2 (CST, D55G8, USA), anti-E-CADHERIN (Proteintech, 20874-1-AP,USA) and anti-GAPDH (Bioworld, AP0066, USA), overnight at 4°C. Subsequently, membranes were incubated with HRP-conjugated affiniPure goat antibodies IgG (BOSTER, China) for 1.5 h. The target bands were analyzed using ECL Super Signal (Pierce, USA).

### Hematoxylin and Eosin (H&E) Staining

Tumor tissues from the control and chrysin groups were fixed in 4% paraformaldehyde for 48 h, embedded in paraffin wax and sliced into 5 μm sections. The slides were then stained with H&E and cancer cell infiltration was determined by observation under a light microscope.

### Animals and Animal Care

For *in vivo* experiments, 17 female nude mice (6–8 weeks old) were utilized to determine the effect of chrysin treatment and *COPB2* KO on tumor growth. The mice were acquired and grouped-housed in the Laboratory Animal Center of Jilin University. All mice were provided *ad libitum* access to standard rodent food and tap water within the laboratory cages, as well as under specific pathogen-free (SPF) conditions. The BGC823, pcDNA3.1-COPB2 and COPB2-KO cell lines (3 × 10^6^) were subcutaneously injected into the left flank of each mouse, and tumors were observed after seven days. The tumor length (L) and width (W) were calculated as L × W^2^/2.

### Statistical Analysis

An unpaired Student’s *t*-test was utilized in the present study. The SPSS 16.0 software (SPSS Inc., Chicago, IL, USA) helped conduct statistical analysis. All data was expressed as mean ± SD. A *p-*value of <0.05 was considered to be statistically significant. The website of http://ualcan.path.uab.edu/index.html was used for The Cancer Genome Atlas (TCGA) analysis. The TargetScan database was used to predict miRNA.

## Results

### Screen of Differentially Expressed Gene of Chrysin-Treated GC Cells

**Figure 1:**
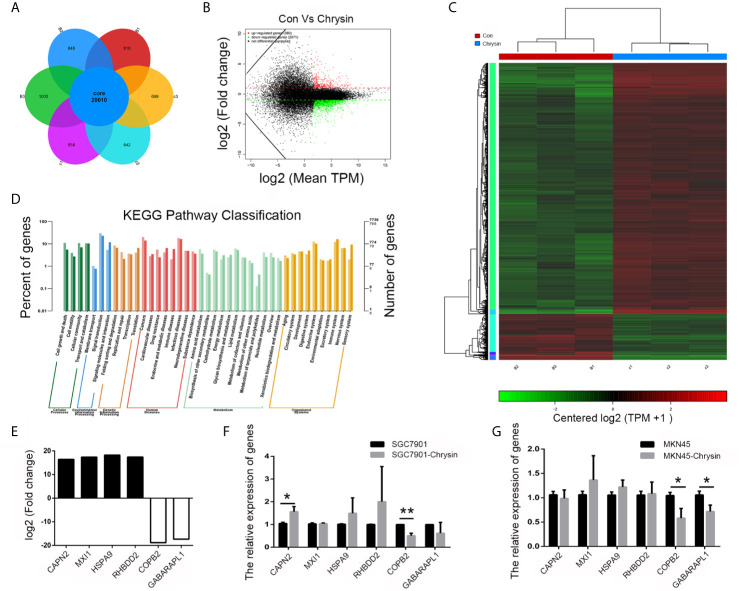
Screening of differentially expressed genes by RNA-seq. Analysis of differential expressed genes after chrysin treatment **(A)**, Identification of different expressed genes **(B)**. The heatmap was drawn to show the differentially expressed genes **(C)**. KEGG pathway of the differentially expressed genes **(D)**. The expression of log2 fold change in six genes **(E)**. Relative expression of *CAPN2*, *MXI1*, *HSPA9*, *RHBDD2*, *COPB2* and *GABAPAPL1* were analyzed by qPCR after Chrysin treatment in SGC7901 **(F)** and MKN45 cells **(G)**. The data are represented as the mean ± SD (n = 3). * (*p <*0.05) and ** (*p <*0.01) indicate statistically significant differences.

In order to analyze gene expression patterns of chrysin treatment in gastric cancer cells, we performed RNA-Seq. Overall, 20,010 genes were identified as core genes (**Figure 1A**). Compared to the control group, 380 genes were significantly up-regulated while 2,071 were significantly down-regulated (**Figure 1B**). Data from heatmap and KEGG pathway suggests that the differentially expressed genes have functions in cell death and growth (**Figures 1C, D**). In order to confirm this data, six genes (*CAPN2*, *MXI1*, *HSPA9*, *RHBDD2*, *COPB2* and *GABAPAPL1*), which were related to cell growth and death, were further validated (**Figure 1E**). The qPCR results indicated that *COPB2* expression was downregulated upon chrysin treatment in SGC7901 (**Figure 1F**) and MKN45 (**Figure 1G**) cells. These results indicate that *COPB2* expression is regulated by chrysin in GC cells.

### Chrysin Increased let-7a and Inhibited *H19* and *COPB2* Expression in GC Cells

**Figure 2:**
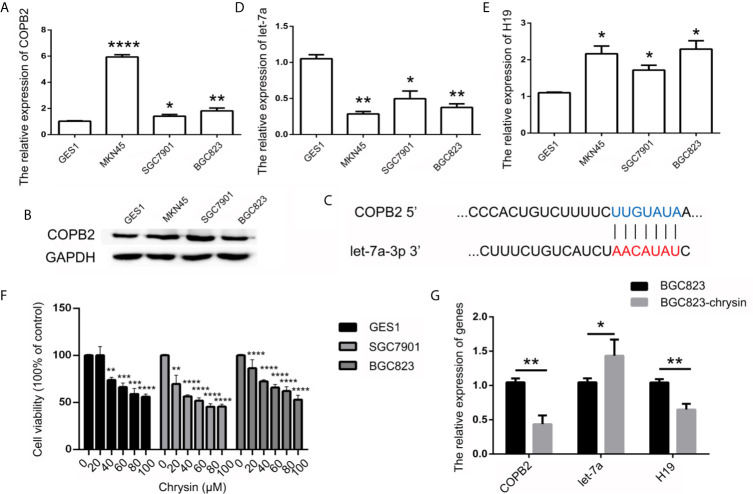
Analysis of *COPB2*, let-7a and *H19* expression pattern. The expression of COPB2 in GC cells using qPCR **(A)** and western blot **(B)**. Schematic representations of *COPB2* and let-7a **(C)**. Relative expression of let-7a in GC ells **(D)**. Relative expression of *H19* in GC ells **(E)**. The cell growth was analyzed by CCK8 assay **(F)**. Relative expression of *COPB2*, let-7a and *H19* was analyzed by qPCR after chrysin treatment in GC cells **(G)**. The data are represented as the mean ± SD (n = 3). * (*p <*0.05), ** (*p <*0.01), *** (*p <*0.001) and **** (*p <*0.0001) indicate statistically significant differences.

**Figure 3:**
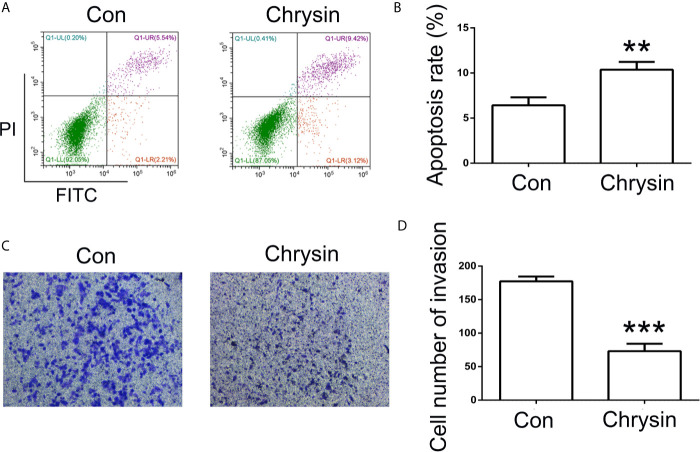
Analysis of cell apoptosis and invasion after chrysin treatment. The cell apoptosis was analyzed between Con and chrysin group **(A)**. Statistical analysis of the percentage of cell apoptosis **(B)**. The cell invasion was analyzed **(C)**. Statistical analysis of the percentage of cell invasion **(D)**. The data are represented as the mean ± SD (n = 3). ** (*p <*0.01) and *** (*p <*0.001) indicate statistically significant differences.

In order to further verify the expression levels of *COPB2* in GC, we utilized the TCGA database. Results indicated increased expression of *COPB2* in primary tumor of the stomach adenocarcinoma (STAD) patients (**Figure S1A**). Compared to GES1 cells, qPCR and western bolt results indicated increased expression of COPB2 in MKN45, SGC7901 and BGC823 cells (**Figures 2A, B**). To investigate which miRNAs were involved in *COPB2* expression, bioinformatics analysis was performed. The database suggested that let-7a targets *COPB2* (**Figures 2C**, **S2**). Furthermore, we analyzed let-7a levels in the TCGA database. Results indicated no differences between normal and tumor tissues (**Figure S1B**). However, qPCR results suggested that let-7a levels were reduced in GC cells, compared to GES1 cells (**Figure 2D**). Considering that let-7a is associated with expression of the lncRNA *H19*, we analyzed the expression pattern of *H19*. The TCGA database indicated increased expression of *H19* among STAD patients (**Figure S1C**). The qPCR results indicated increased expression of *H19* in GC cells (**Figure 2E**). DNA methylation results indicated the hypo-methylation profile of *H19* DMR in GC cells (**Figure S3**). The cell growth was analyzed after chrysin treatment. The CCK8 results indicated that 40 μM was the optimal dose for subsequent experiments (**Figure 2F**). Moreover, qPCR results indicated increased expression of let-7a, as well as reduced expression of *H19* after chrysin treatment in GC cells (**Figure 2G**). Besides, chrysin was able to induce cell apoptosis, as well as inhibit cell migration and invasion in GC cells (**Figures 3**, **S4**). These results indicate that chrysin has an anti-cancer role and regulates expression of *COPB2*, *H19* and let-7a in GC cells.

### *H19*/let-7a Regulate *COPB2* Expression

**Figure 4:**
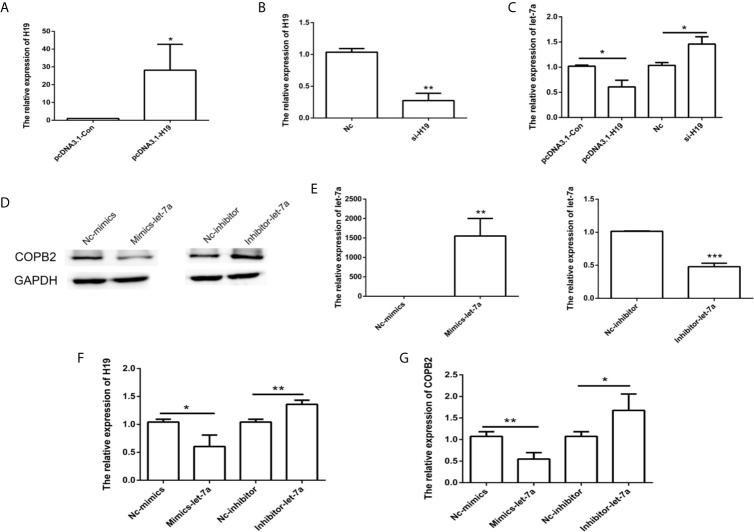
Analysis of *COPB2* expression pattern through *H19*/let-7a in GC cells. Relative expression of *H19* and let-7a in pcDNA3.1-Con, pcDNA3.1-H19, Nc and si-H19 group using qPCR **(A–C)**. Expression of COPB2 protein in Nc-mimics, mimics-let-7a, Nc-inhibitor and inhibitor-let-7a group using Western blot **(D)**. Relative expression of let-7a, *H19* and *COPB2* in Nc-mimics, mimics-let-7a, Nc-inhibitor and inhibitor-let-7a group using qPCR **(E–G)**. The data are represented as the mean ± SD (n = 3). * (*p <*0.05), ** (*p <*0.01) and *** (*p <*0.001) indicate statistically significant differences.

Considering that *H19* has a role in let-7a expression, we analyzed the effect of *H19* knockdown and overexpression in GC cells. The results indicated reduced expression of let-7a in the *H19* overexpression group, as well as overexpression of let-7a in the *H19* knockdown group (**Figures 4A–C**). In order to investigate the expression pattern of let-7a, we utilized miRNA mimics and inhibitors. The qPCR and western blot results demonstrated that let-7a mimics led to suppressed expression of *COPB2* (**Figures 4D–G**). These results confirm that *H19* acts as a sponge that competes with let-7a, thus regulating *COPB2* expression.

### Reduced Expression of *COPB2* Induced Cell Apoptosis and Inhibited Cell Invasion

**Figure 5:**
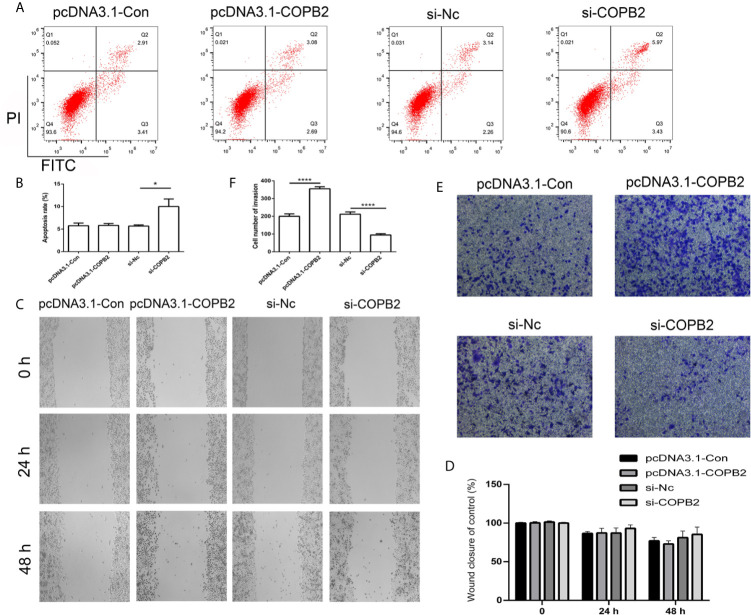
Analysis of cell apoptosis, migration and invasion after knockdown and overexpression of *COPB2*. Cell apoptosis was analyzed in the pcDNA3.1-Con, pcDNA3.1-COPB2, si-Nc, and si-COPB2 group **(A, B)**. Cell migration was analyzed after si-COPB2 and pcDNA3.1-COPB2 were transfected **(C, D)**. The cell invasion was analyzed in pcDNA3.1-Con, pcDNA3.1-COPB2, si-Nc, and si-COPB2 group **(E, F)**. Statistical analysis of the percentage of cell invasion **(D)**. The data are represented as the mean ± SD (n = 3). * (*p <*0.05) and **** (*p <*0.0001) indicate statistically significant differences.

In order to analyze whether *COPB2* expression has an effect on cell death and growth, we transfected knockdown and overexpression vector of *COPB2* into GC cells. The CCK8 data demonstrates that reduced expression of *COPB2* and increased expression of let-7a inhibits cell growth (**Figure S5**). Moreover, our results suggested that reduced expression of *COPB2* induced cellular apoptosis (**Figures 5A, B**). In order to validate this, we analyzed markers of cell apoptosis. The results showed that increased expression of p53 was observed in the *COPB2* knockdown group (**Figure S6**). In addition, *COPB2* expression did not have an effect on cell migration in GC cells (**Figures 5C, D**). However, our data shows that reduced expression of *COPB2* inhibited cell invasion (**Figures 5E, F**). Next, we investigated the effect of chrysin about cell migration and invasion in the COPB2 overexpression group. This result demonstrated that chrysin induced cell apoptosis and inhibited cell migration and invasion (**Figures S7**, **S8**). In order to validate this finding, the overexpression and knockdown of *H19* and let-7a were used to analyze cell apoptosis. The results demonstrated that reduced expression of *H19* and increased expression of let-7a induced cell apoptosis (**Figure S9**). Overall, these results suggest that *COPB2* has a role in cell apoptosis and invasion.

### Loss Expression of *COPB2* Inhibited Tumor Growth *In Vivo*

**Figure 6:**
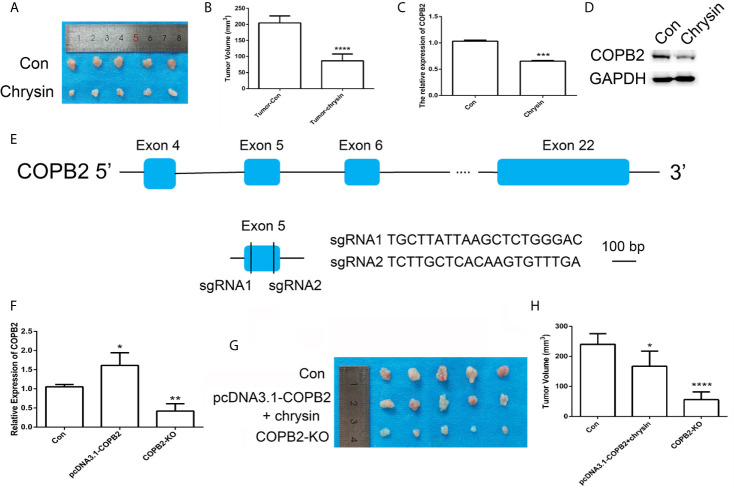
CRISPR/Cas9-mediated gene targeting of *COPB2*. Morphological observation of mouse tumor tissue **(A)**. Analysis of tumor volume **(B)**. The expression pattern of *COPB2* in tumor of mice after chrysin treatment **(C, D)**. Schematic diagram of sgRNAs targeting the *COPB2* gene loci **(E)**. The expression of *COPB2* using qPCR **(F)**. The tumor morphology **(G)** and volume **(H)**. Blue indicated *COPB2*. * (*p <* 0.05), ** (*p <* 0.01), *** (*p <* 0.001) **** and (*p <* 0.0001) indicate statistically significant differences.

In order to assess the anti-cancer effect of chrysin *in vivo*, we utilized nude mice. The BGC823 cells were injected into nude mice and after seven days, mice were treated with chrysin (20 mg/kg) for two weeks. The results showed that chrysin is able to inhibit tumor growth and *COPB2* expression *in vivo* (**Figures 6A–D**). The H&E staining results confirm this data (**Figure S10**). Moreover, qPCR result indicated that chrysin inhibited the expression of *H19*, and increased let-7a *in vivo* (**Figure S11**). In order to determine the effect of loss expression of *COPB2 in vivo*, CRISPR/Cas9 system was used to edit the *COPB2* exon 5 (**Figure 6E**). The qPCR data showed lower expression of *COPB2* in the *COPB2* KO group compared to the control group (**Figure 6F**). Moreover, chrysin treatment reduced *COPB2* expression in overexpression and KO of *COPB2* cells (**Figure S12**). To further confirm the effect of *COPB2* expression, *COPB2* KO cells were injected into nude mice. Results suggested that loss of expression of *COPB2* inhibited tumor growth (**Figures 6G, H**). Overall, these results indicate that reduced expression of *COPB2* leads to anti-tumor effects.

## Discussion

Chrysin, a natural medicine, has anti-inflammatory and anti-cancer function and has been used to treat degenerative disorders and cancers in several Asian countries (26, 27). In this study, chrysin was used to treat GC cells in order to evaluate its effect on cellular apoptosis, growth, migration and invasion. Previous reports have indicated that chrysin induces cell apoptosis and inhibits cell growth, migration and invasion in glioblastoma cells (28), which was by validated by our results, which showed that chrysin has anti-cancer effects in SGC7901, MKN45 and BGC823 cells. Moreover, chrysin was found to increase expression of miR-9 and let-7a in GC cells, in accordance with previous data (29). Interestingly, our previous data suggested that chrysin inhibited cell migration and invasion in MKN45 cells through TET1, which regulates global DNA methylation (30). Previous reports have indicated abnormal DNA methylation in GC (31). Herein, our results suggested that *H19* DMR is hypomethylated in SGC7901 and BGC823 cell lines, which is related to increased expression of *H19*. In addition, chrysin functions to regulate the expression of *H19*, let-7a and *COPB2*. Furthermore, chrysin inhibited cell invasion which was overexpression of *COPB2*.

Recently, lncRNAs and miRNAs have been demonstrated to have roles in development of different types of cancer, including HCC (18). There is evidence that reduced expression of lncRNA *H19* leads to inhibition of tumor growth in breast cancer, bladder cancer and colorectal cancer (32). Moreover, *H19*, as a competitive endogenous RNA, is associated with miRNAs, such as miR-29 and let-7 (16, 33). Our results indicated that *H19* and let-7a have competitive regulation in GC cells, which has also been confirmed in a previous report (17). Emerging evidence suggested that loss of expression of let-7 correlates with poor prognosis in various cancer (34). Our data showed that chrysin increased expression of let-7a and inhibited cell migration and invasion. Previous reports indicated that silencing of let-7, which targets MDM4, promotes cell proliferation, migration and invasion (35). Further, reduced expression of *H19* and increased let-7a expression induced cell apoptosis in GC cells, which validated the previous report (36). These results suggested that the expression of *H19* and let-7a is involved in cell apoptosis, growth and invasion of GC cells.

**Figure 7:**
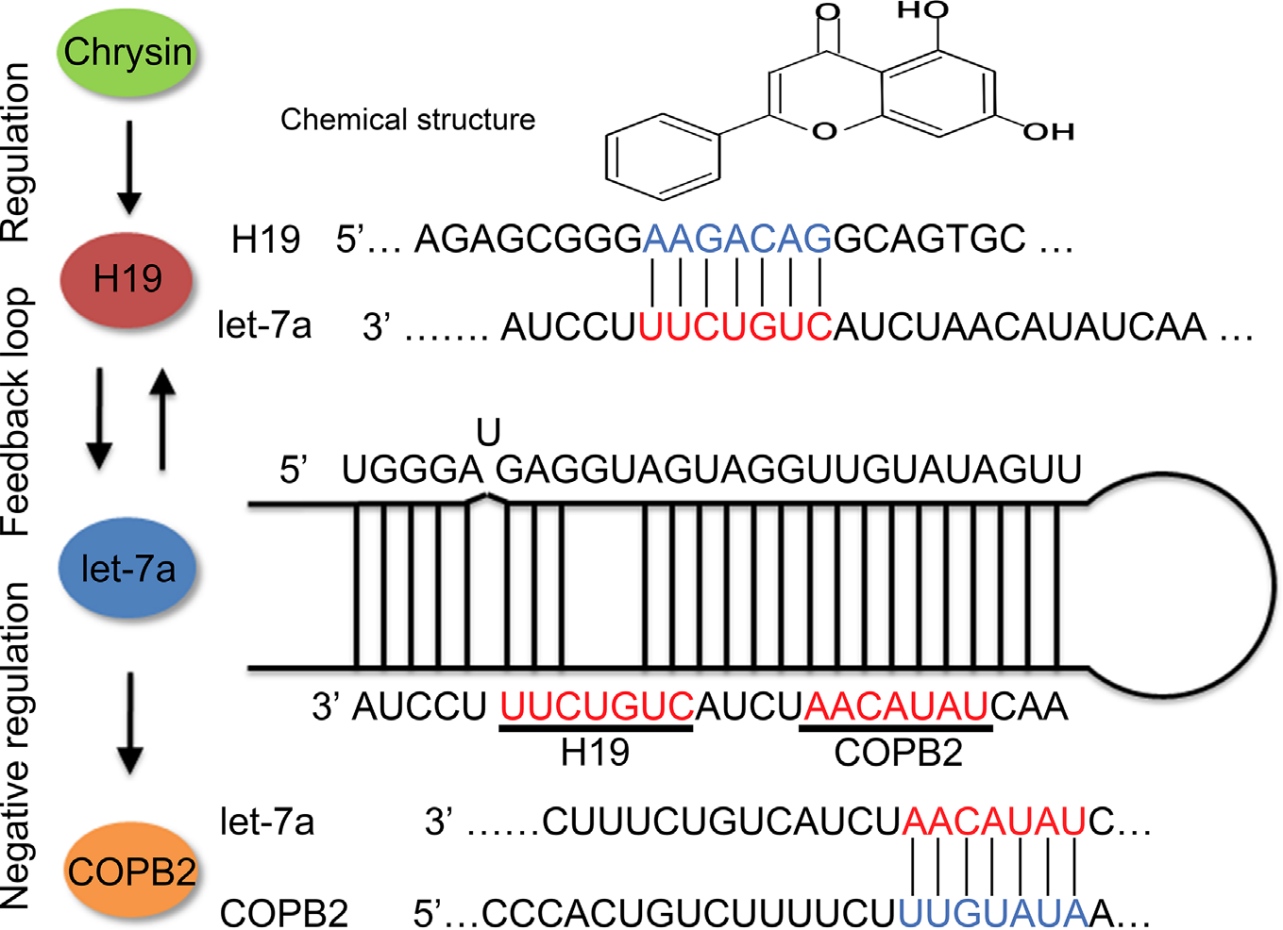
Schematic diagram of *H19*/let-7a regulate *COPB2* expression.

Previous reports indicated that let-7f targets HMGA2 in thyroid cancer (37). Compared to let-7f, *IL-6* and *CKIP-1* were reported to be targets of let-7a (38, 39). Our results suggest that let-7a targets *COPB2*, which leads to differential expression after chrysin treatment in GC cells. A previous study indicated that the expression of *COPB2* is associated with cell growth, apoptosis, migration and invasion, functioning through a miR-216a manner, in lung cancer (8). Our results suggested that expression of *COPB2* was regulated by *H19*/let-7 axis in GC cells (**Figure 7**). Moreover, reduced expression of *COPB2* induced cellular apoptosis and inhibited cell growth in prostate cancer (40). Our data showed reduced expression of *COPB2* increased p53 and E-cadherin expression. These results indicated that reduced expression of *COPB2* induced cell apoptosis and inhibited invasion through *H19*/let-7a.

In order to confirm the effect of *COPB2 in vivo*, a xenograft model using nude mice was established. The *in vivo* results suggested that chrysin led to reduced expression of *COPB2*, which further confirms our data in GC cells. Furthermore, chrysin inhibited tumor growth *in vivo*, which is in accordance with a previous report in melanomas (41). These data indicate that chrysin can regulate *COPB2* expression, which inhibits tumor growth *in vivo*. In order to further analyze the putative effect of *COPB2*, the KO and overexpression *COPB2* cell line were injected into nude mice. As a powerful gene editing tool, the CRISPR/Cas9 system was wildly used *in vitro* and *in vivo*. Our results indicated that *COPB2* KO suppressed tumor growth.

In summary, *COPB2* is a differentially expressed gene that was identified after chrysin treatment in GC cells. Our data indicated that *COPB2* is regulated by let-7a, which acts as a molecular sponge of *H19*. Moreover, reduced expression of *COPB2* induced cellular apoptosis and inhibited cell growth and invasion. Therefore, this present study revealed that *COPB2* is a potential molecular targeted therapy in GC.

## Data Availability Statement

The datasets presented in this study can be found in online repositories. The names of the repository/repositories and accession number(s) can be found in the article/**Supplementary Material**.

## Ethics Statement

The animal study was reviewed and approved by Laboratory Animal Center of Jilin University.

## Author Contributions

DW designed the experiments and wrote the manuscript. LC, QL, HH, and CL performed cell experiment and gene expression analysis. TW and ZJ contributed reagents and materials. YG and CL carried out animal experiment. DW analyzed the data and prepared figures. All authors contributed to the article and approved the submitted version.

## Funding

This work was supported by the Jilin Health Commission Program under Grant 2020J05S, the Fundamental Research Funds for the Central Universities under Grant 2019JCKT-70, the Jilin Education Department Program under Grant JJKH20200950KJ, and the Jilin Scientific and Technological Development Program under Grant 20190103071JH.

## Conflict of Interest

The authors declare that the research was conducted in the absence of any commercial or financial relationships that could be construed as a potential conflict of interest.

## Supplementary Material

Supplementary Figure 1 Analysis of *COPB2*, let-7a and *H19* in TCGA database. Effect of *COPB2* **(A)**, let-7a **(B)** and *H19* **(C)** expression on GC patient survival in TCGA database.

Supplementary Figure 2 Screen of miRNA which regulated by COPB2.

Supplementary Figure 3 The DNA methylation status of *H19* DMR in MGC823 and SGC7901 using BSP.

Supplementary Figure 4 Analysis of cell migration after chrysin treatment. The cell migration was analyzed between Con and chrysin group **(A)**. Statistical analysis of the percentage of cell migration **(B)**. **** (*p* < 0.0001) indicate statistically significant differences.

Supplementary Figure 5 The CCK8 assay results in overexpression and knockdown of *COPB2*. The CCK8 assay in GES1 cells after chrysin treatment **(A)**. The CCK8 assay in pcDNA3.1-Con, pcDNA3.1-H19, Nc and si-H19 group **(B)**. The CCK8 assay in Nc-mimics, mimics-let-7a, Nc-inhibitor and inhibitor-let-7a group **(C)**. The mRNA expression of *COPB2* in the pcDNA3.1-Con, pcDNA3.1-COPB2, si-Nc, si-COPB2 group using qPCR **(D)**. The CCK8 assay in pcDNA3.1-Con, pcDNA3.1-COPB2, si-Nc, and si-COPB2 group **(E)**. ** (*p* < 0.01), *** (*p* < 0.001) and **** (*p* < 0.0001) indicate statistically significant differences.

Supplementary Figure 6 Analysis of cell apoptosis markers. The protein expression of COPB2, BAX, BCL2, P53 and E-Cadherin were analyzed using western blot **(A)**. The relative mRNA expression of *BAX*, *BCL2* and *TP53* were analyzed using qPCR **(B)**. * (*p* < 0.05) and ** (*p* < 0.01) indicate statistically significant differences.

Supplementary Figure 7 Cell apoptosis analysis. The cell apoptosis was analyzed after chrysin treatment in pcDNA3.1-COPB2 group **(A, B)**. *** (*p* < 0.001) indicate statistically significant differences.

Supplementary Figure 8 The cell migration and invasion analysis. The cell migration was analyzed after chrysin treatment in pcDNA3.1-COPB2 group **(A, B)**. Cell invasion was analyzed after chrysin treatment in the pcDNA3.1-COPB2 group **(C, D)**. * (*p* < 0.05) and ** (*p* < 0.01) indicate statistically significant differences.

Supplementary Figure 9 Cell apoptosis and cell growth analysis. The cell apoptosis was analyzed in pcDNA3.1-Con, pcDNA3.1-H19, Nc and si-H19 group **(A, B)**. Cell apoptosis was analyzed in the Nc-mimics, mimics-let-7a, Nc-inhibitor and inhibitor-let-7a group **(C, D)**. ** (*p* < 0.01) and *** (*p* < 0.001) indicate statistically significant differences.

Supplementary Figure 10 Histopathological observation of tumor tissue.

Supplementary Figure 11 Analysis of *H19* and let-7a levels after chrysin treatment using qPCR *in vivo*. * (*p* < 0.05) and **** (*p* < 0.0001) indicate statistically significant differences.

Supplementary Figure 12 Analysis of chrysin treatment in overexpression and KO of *COPB2*. The genes expression in pcDNA3.1-COPB2 **(A)** and COPB2-KO **(B)** group after chrysin treatment using qPCR. The expression of COPB2 and P53 in pcDNA3.1-COPB2 **(C)** and COPB2-KO **(D)** group after chrysin treatment using western blot. * (*p* < 0.05) and ** (*p* < 0.01) indicate statistically significant differences.
