# Supplementary figures and images for: Chrysin Induced Cell Apoptosis Through H19/let-7a/COPB2 Axis in Gastric Cancer Cells and Inhibited Tumor Growth
Source: Front Oncol. 2021 Jun 3;11:651644. doi: 10.3389/fonc.2021.651644 (PMC8209501; doi:10.3389/fonc.2021.651644)

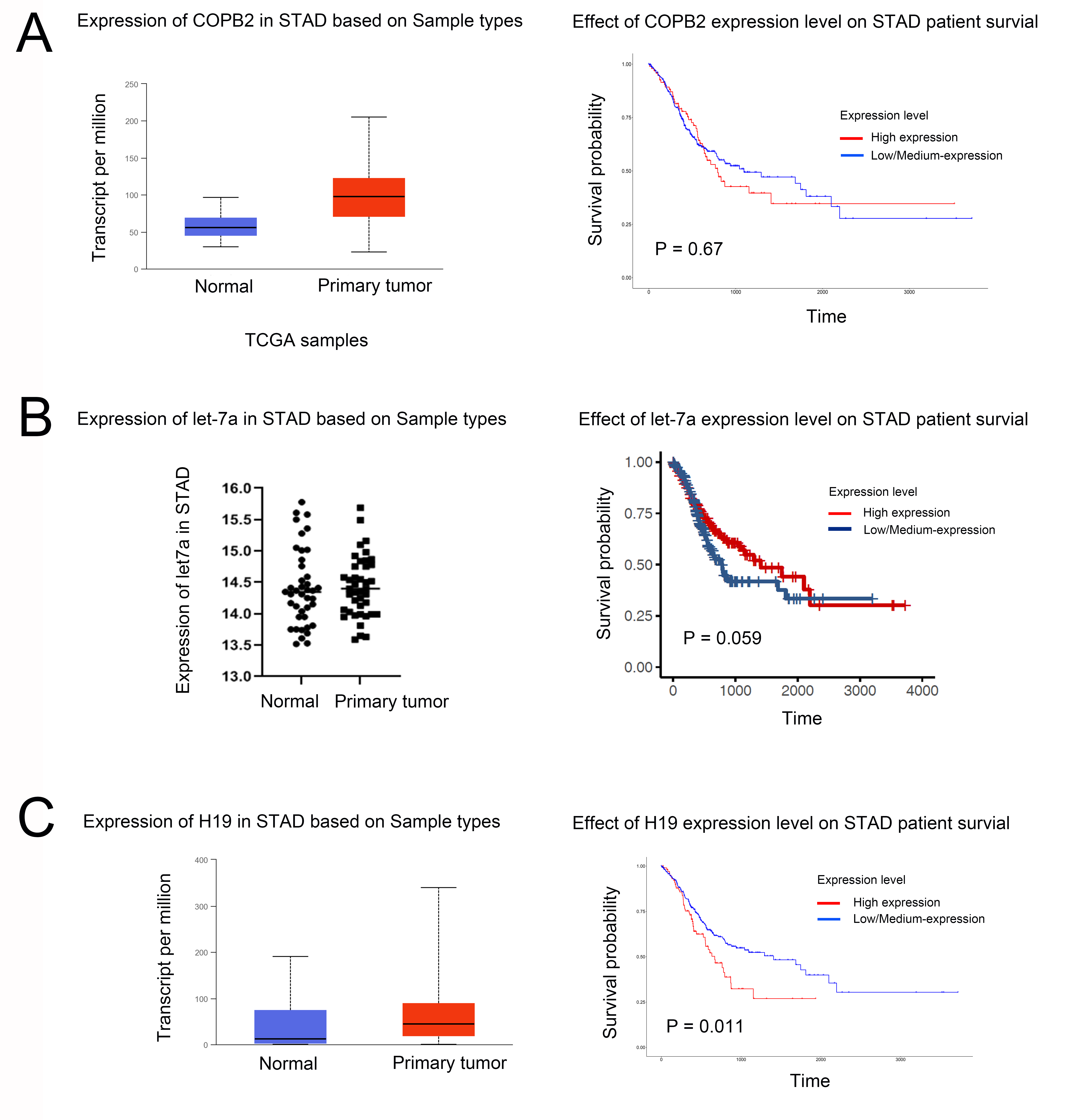

Supplement: Supplementary Figure 1 — Analysis of COPB2, let-7a and H19 in TCGA database. Effect of COPB2 (A), let-7a (B) and H19 (C) expression on GC patient survival in TCGA database. [file Image_1.tif]

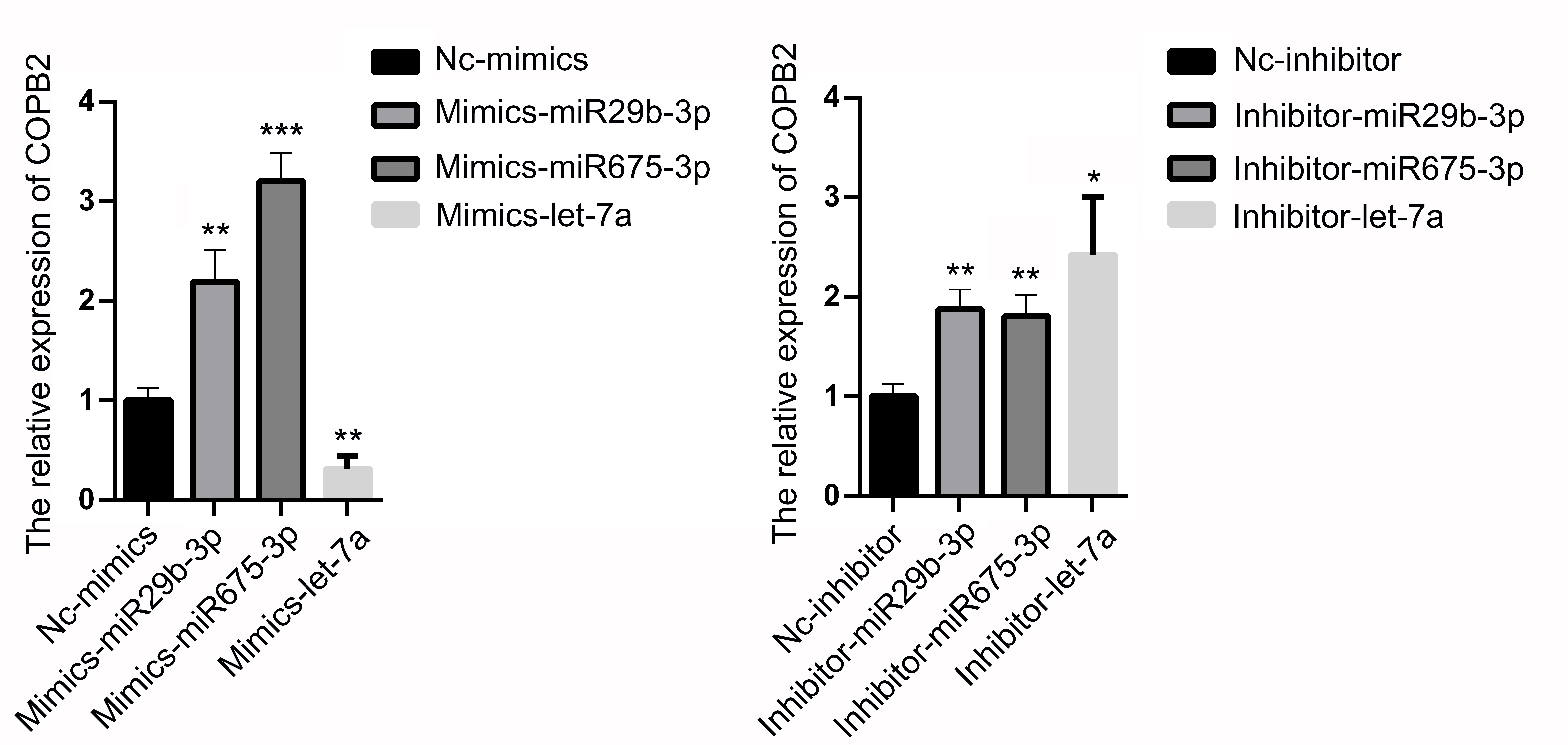

Supplement: Supplementary Figure 2 — Screen of miRNA which regulated by COPB2. [file Image_2.tif]

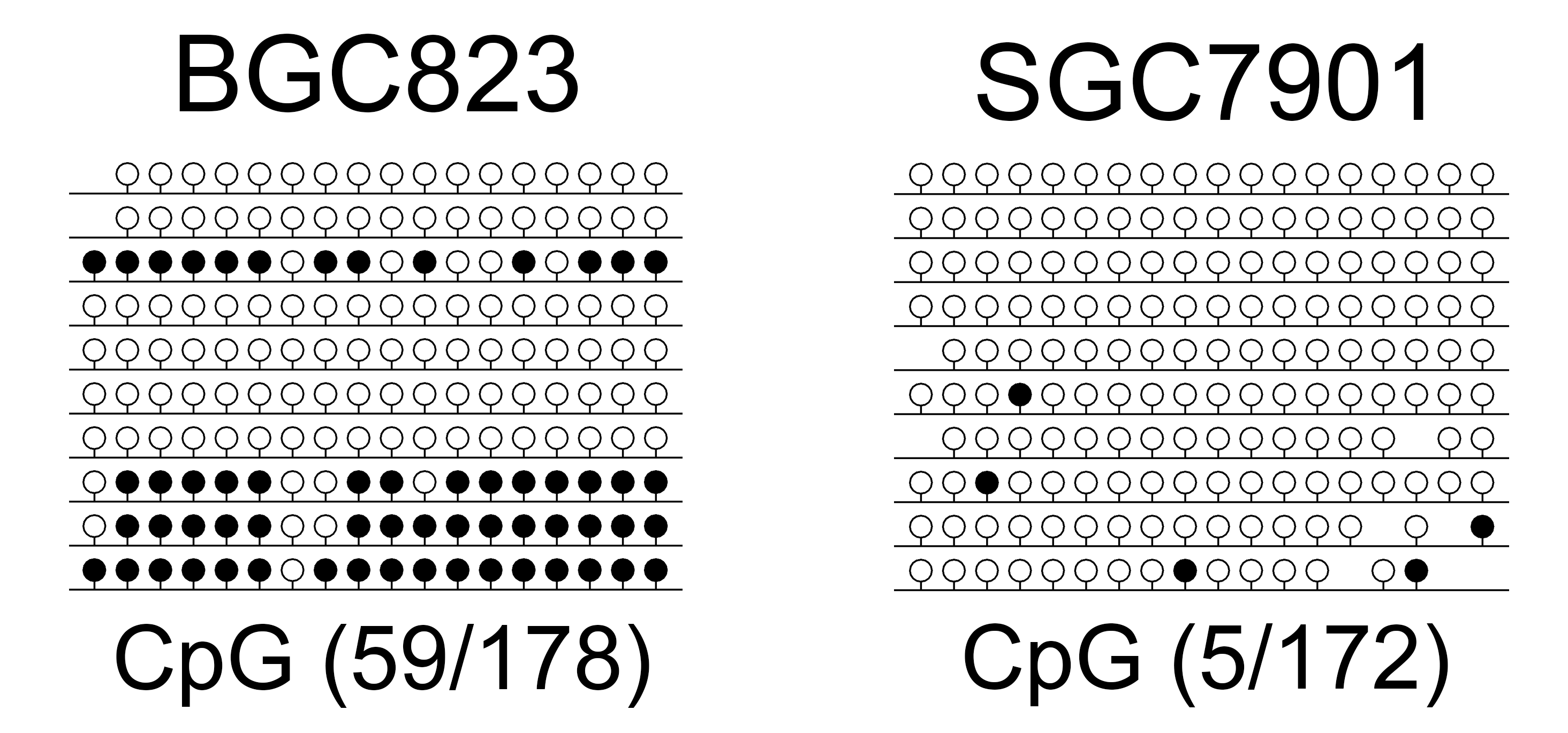

Supplement: Supplementary Figure 3 — The DNA methylation status of H19 DMR in MGC823 and SGC7901 using BSP. [file Image_3.tif]

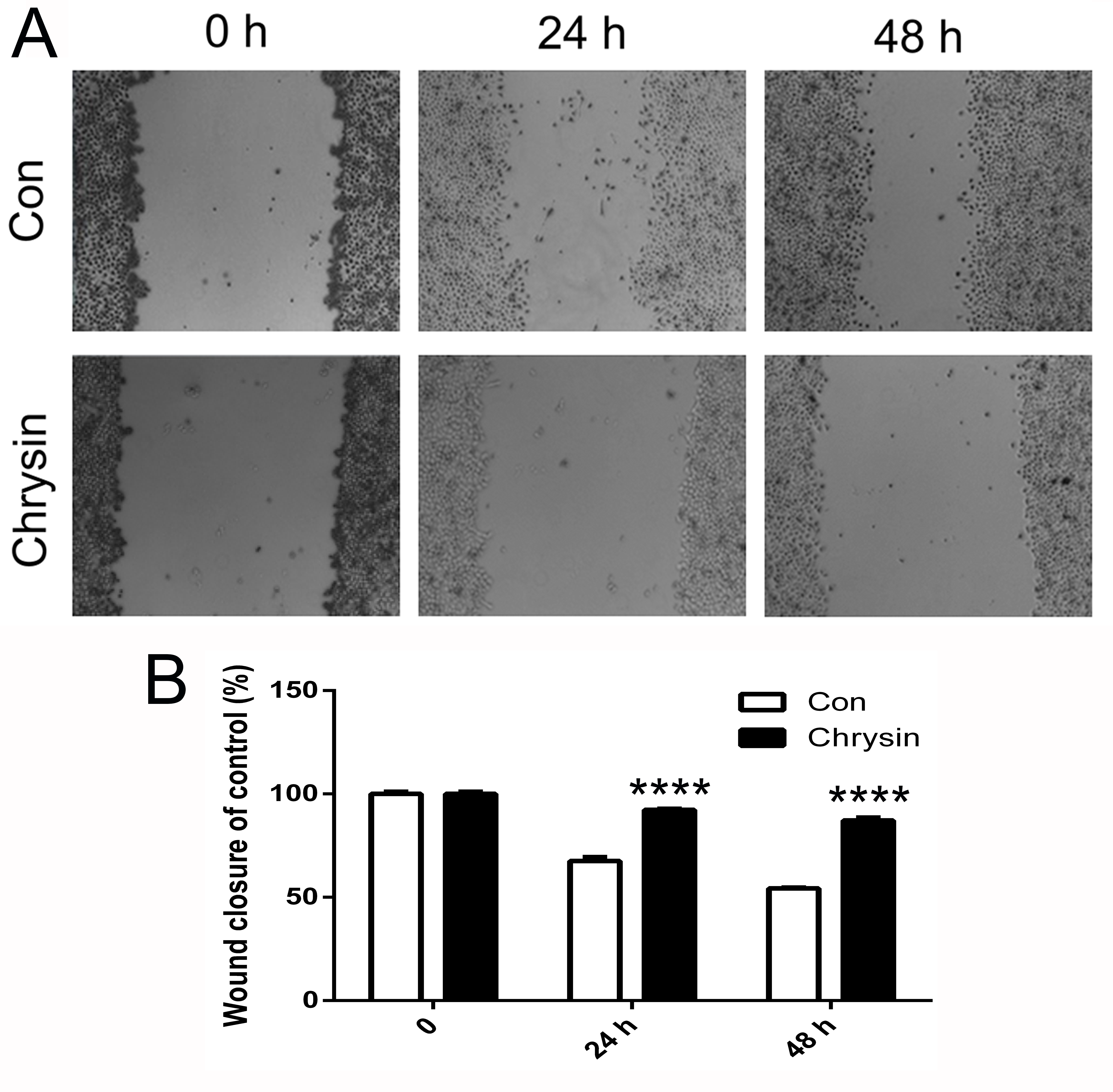

Supplement: Supplementary Figure 4 — Analysis of cell migration after chrysin treatment. The cell migration was analyzed between Con and chrysin group (A). Statistical analysis of the percentage of cell migration (B). **** (p < 0.0001) indicate statistically significant differences. [file Image_4.tif]

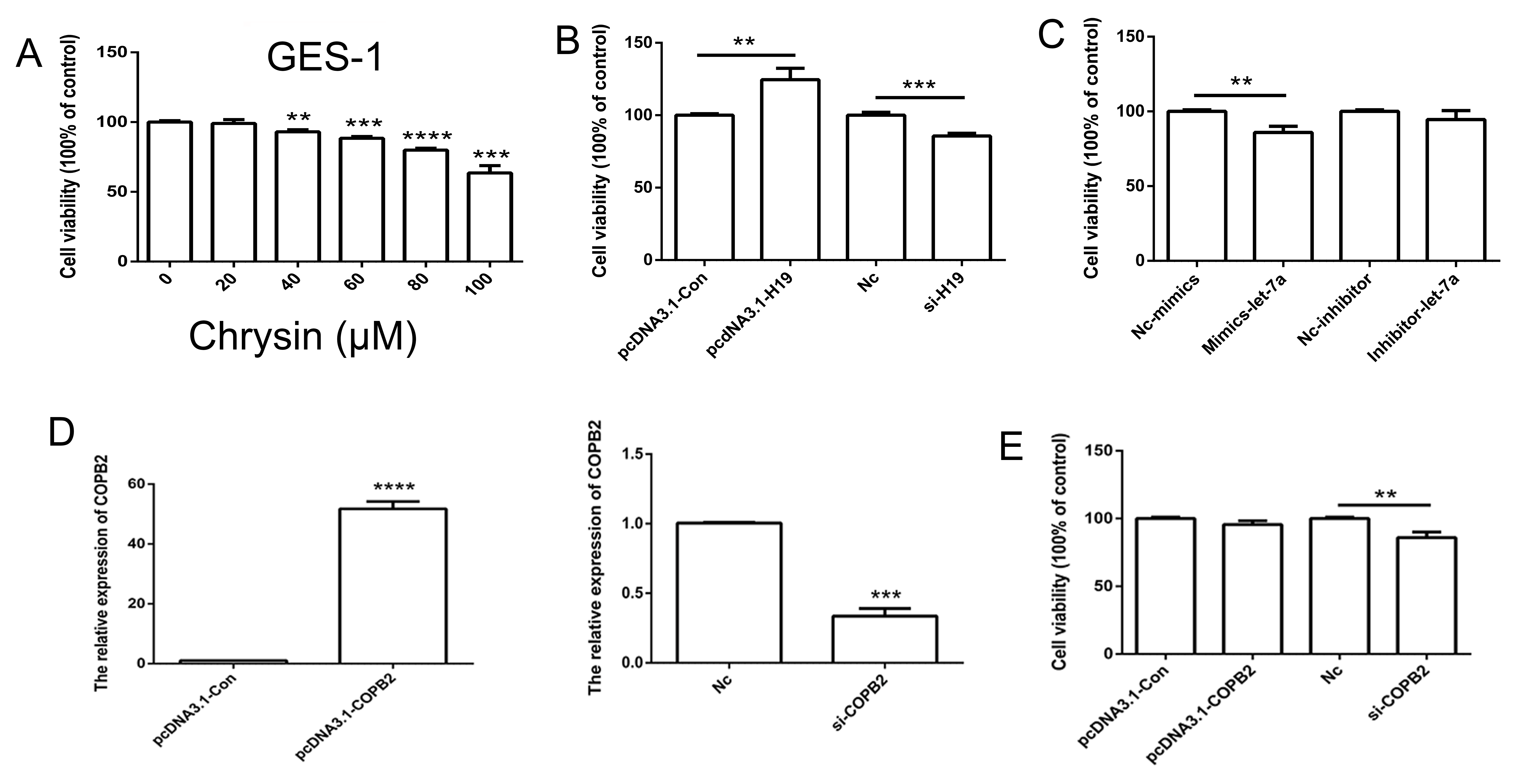

Supplement: Supplementary Figure 5 — The CCK8 assay results in overexpression and knockdown of COPB2. The CCK8 assay in GES1 cells after chrysin treatment (A). The CCK8 assay in pcDNA3.1-Con, pcDNA3.1-H19, Nc and si-H19 group (B). The CCK8 assay in Nc-mimics, mimics-let-7a, Nc-inhibitor and inhibitor-let-7a group (C). The mRNA expression of COPB2 in the pcDNA3.1-Con, pcDNA3.1-COPB2, si-Nc, si-COPB2 group using qPCR (D). The CCK8 assay in pcDNA3.1-Con, pcDNA3.1-COPB2, si-Nc, and si-COPB2 group (E). ** (p < 0.01), *** (p < 0.001) and **** (p < 0.0001) indicate statistically significant differences. [file Image_5.tif]

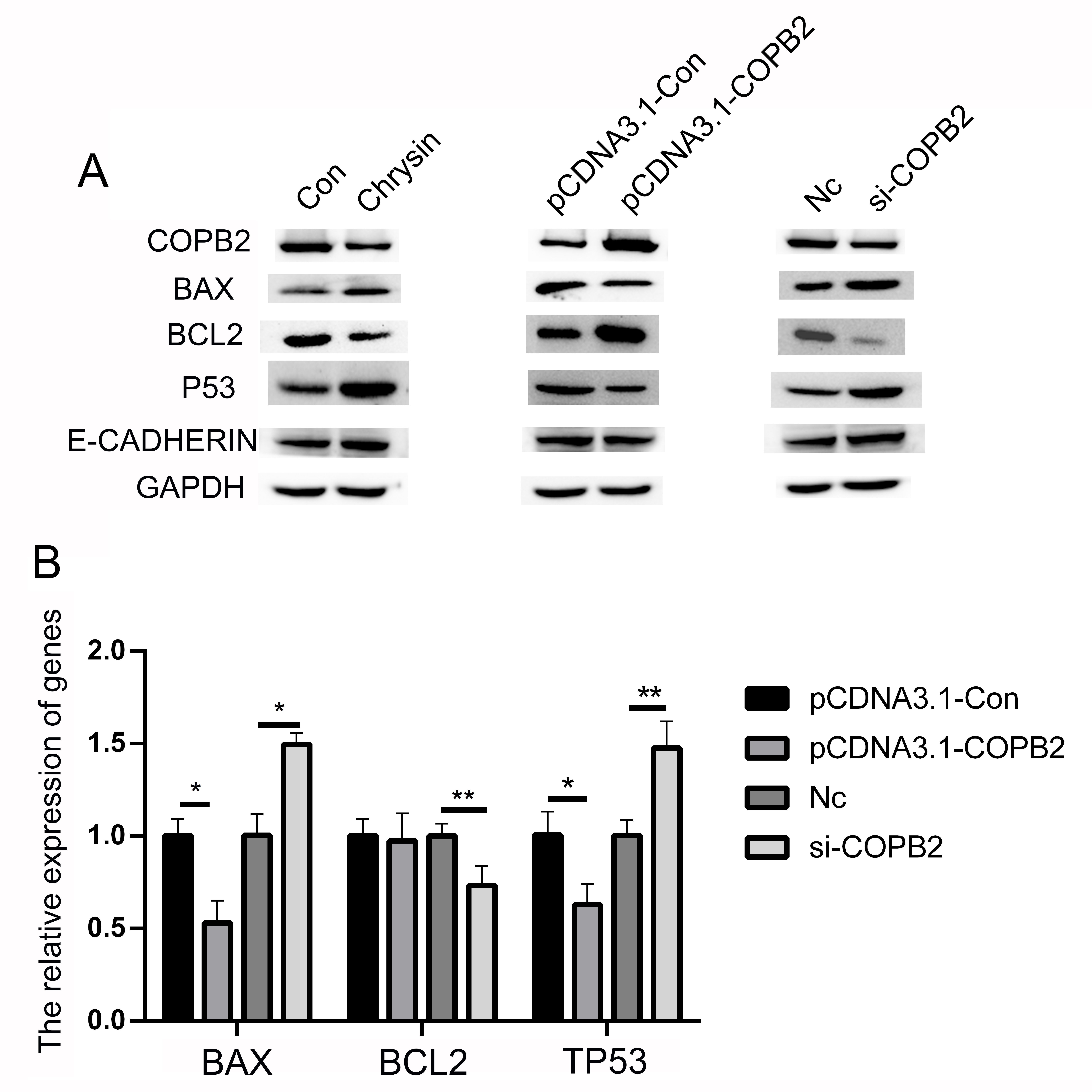

Supplement: Supplementary Figure 6 — Analysis of cell apoptosis markers. The protein expression of COPB2, BAX, BCL2, P53 and E-Cadherin were analyzed using western blot (A). The relative mRNA expression of BAX, BCL2 and TP53 were analyzed using qPCR (B). * (p < 0.05) and ** (p < 0.01) indicate statistically significant differences. [file Image_6.tif]

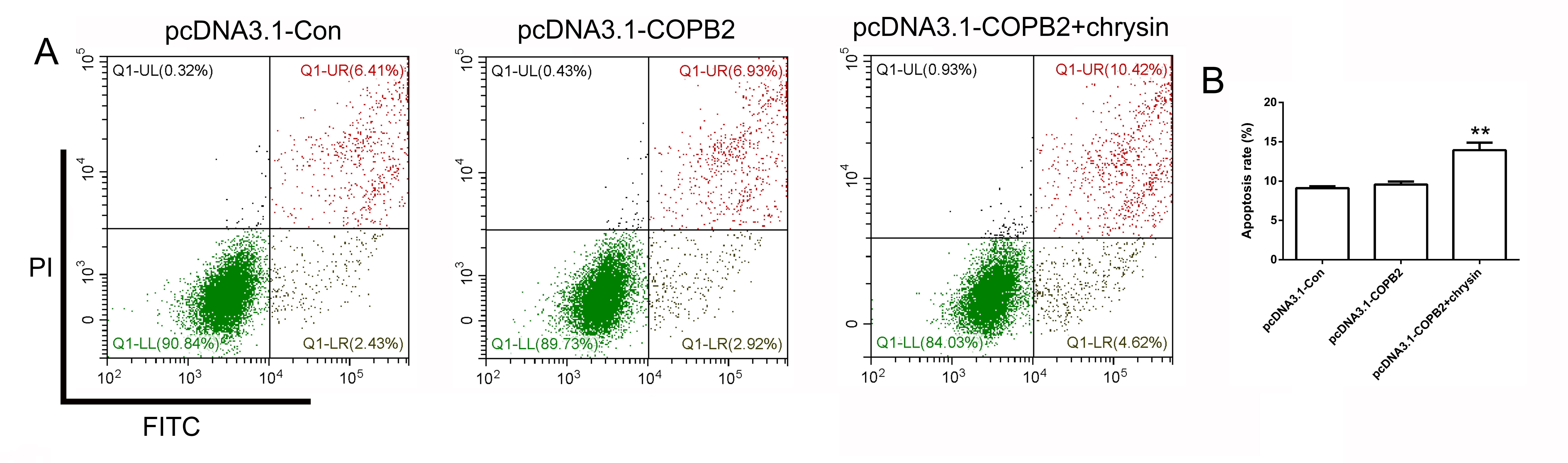

Supplement: Supplementary Figure 7 — Cell apoptosis analysis. The cell apoptosis was analyzed after chrysin treatment in pcDNA3.1-COPB2 group (A, B). *** (p < 0.001) indicate statistically significant differences. [file Image_7.tif]

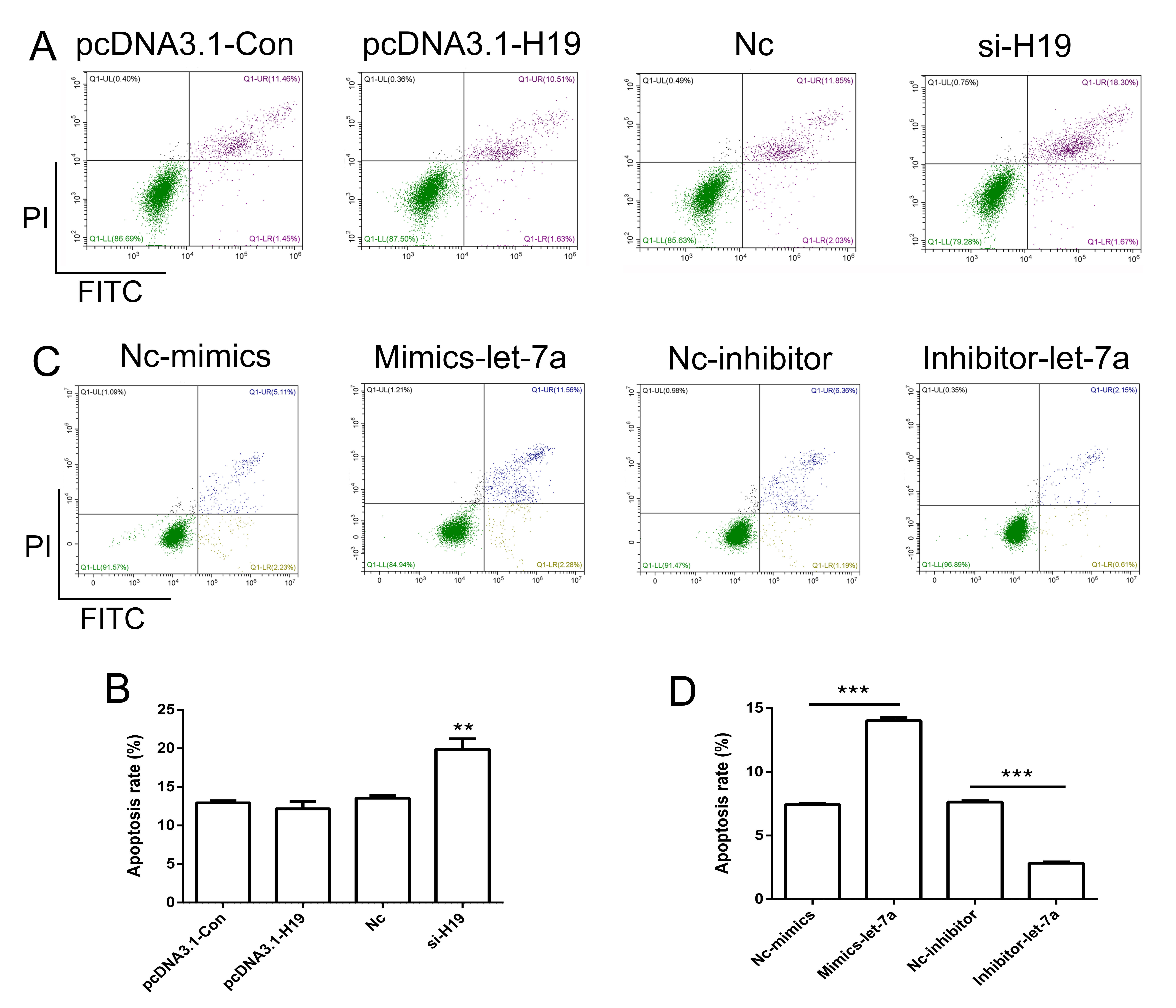

Supplement: Supplementary Figure 9 — Cell apoptosis and cell growth analysis. The cell apoptosis was analyzed in pcDNA3.1-Con, pcDNA3.1-H19, Nc and si-H19 group (A, B). Cell apoptosis was analyzed in the Nc-mimics, mimics-let-7a, Nc-inhibitor and inhibitor-let-7a group (C, D). ** (p < 0.01) and *** (p < 0.001) indicate statistically significant differences. [file Image_9.tif]

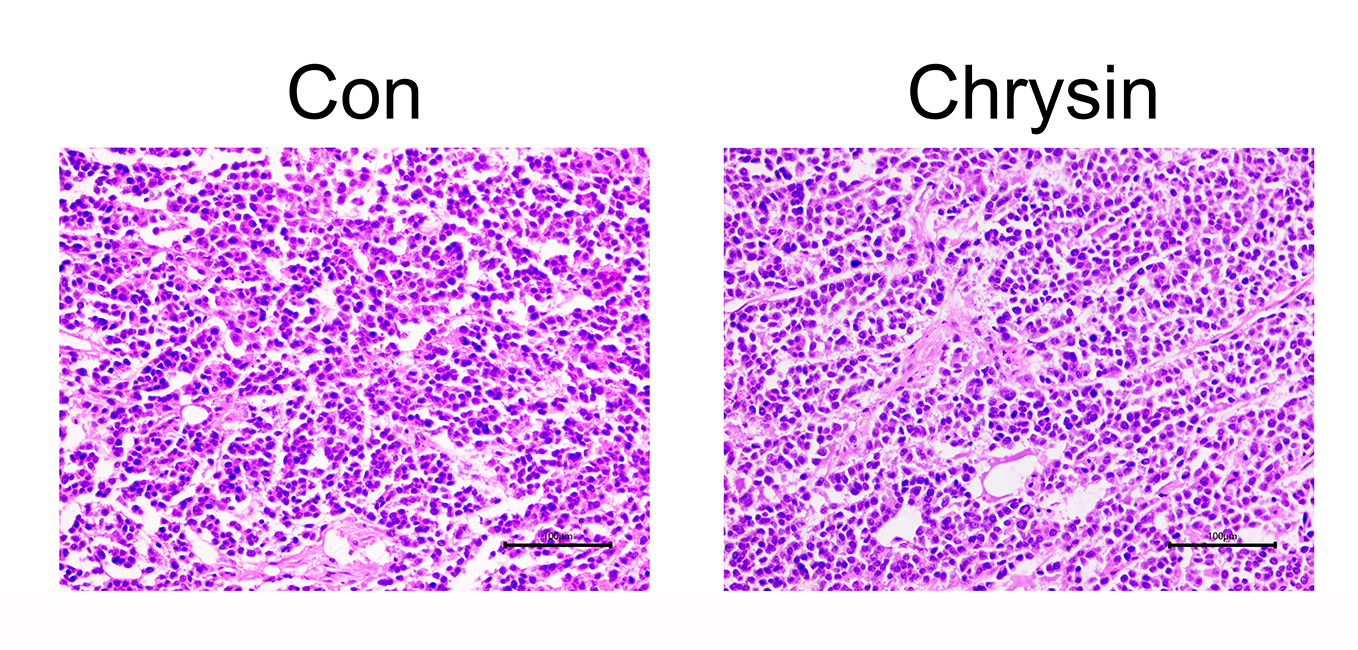

Supplement: Supplementary Figure 10 — Histopathological observation of tumor tissue. [file Image_10.tif]

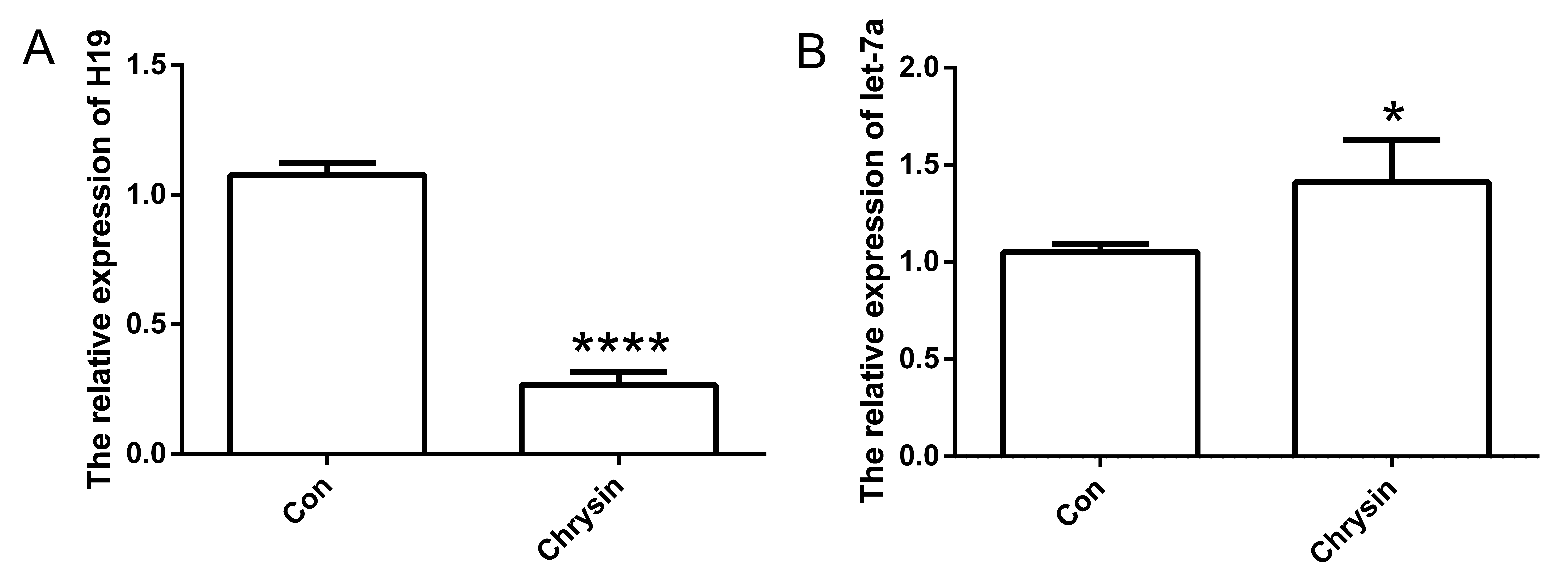

Supplement: Supplementary Figure 11 — Analysis of H19 and let-7a levels after chrysin treatment using qPCR in vivo. * (p < 0.05) and **** (p < 0.0001) indicate statistically significant differences. [file Image_11.tif]
