# Supplementary material for: Chrysin Induced Cell Apoptosis Through H19/let-7a/COPB2 Axis in Gastric Cancer Cells and Inhibited Tumor Growth
Source: Front Oncol. 2021 Jun 3;11:651644. doi: 10.3389/fonc.2021.651644 (PMC8209501; doi:10.3389/fonc.2021.651644)
Supplement: Supplementary file 13 [file Table_1.docx]

**Table**

Table S1 Sequence of siRNA

| siRNA | Sequences（5’→3’） |
| --- | --- |
| *siH19* | GCGGGTCTGTTTCTTTACT |

Table S2 Sequence of sgRNA

| SgRNAs |  | | Sequences (5’→3’) | PAM | | |
| --- | --- | --- | --- | --- | --- | --- |
| SgRNA1 | |  | F: TGCTTATTAAGCTCTGGGAC  R: GTCCCAGAGCTTAATAAGCA | | TGG |  |
| SgRNA2 | |  | F: TCTTGCTCACAAGTGTTTGA  R: TCAAACACTTGTGAGCAAGA | | AGG |  |
| COPB2  Identification | |  | F: AATGGCAGTGTGTGTGTTTGG  R: GCTCTCAAGCCGGTAGGTG | |  |  |

Table S3 Primers for BSP analysis

| Genes | | Annealing (°C) | | Primer sequences (5’→3’) |
| --- | --- | --- | --- | --- |
| H19 DMR  Outer  Inner | 58  58 | | F：TTTTTGGTAGGTATAGAGTT  R：AAACCATAACACTAAAACCC  F: TGTATAGTATATGGGTATTTTTGGAGGTTT  R: TCCCATAAATATCCTATTCCCAAATAACC | |

Table S4 Primers for qPCR analysis

| Genes | Annealing (°C) | | Primer sequences (5’→3’) |
| --- | --- | --- | --- |
| *COPB2* | | 60 | F: CACTTGGACAGGACGATGTAT  R: ACTGAAACCAGCAGGGAATAG |
| *miR-let7a-3p* | | 60 | F:ACACTCCAGCTGGGCTATACAATCTACTG  R: TGGTGTCGTGGAGTCG  RT:CTCAACTGGTGTCGTGGAGTCGGCAATTCAGTTGAGGAAAGACA |
| *H19* | | 60 | F: TTTAGTAGCAGGCACAGGGG  R: CTCCTTGCTGCGCAATGTC |
| *GABARAPL1* | | 60 | F: CCCTCCCTTGGTTATCATCCA  R: ACTCCCACCCCACAAAATCC |
| *CAPN2* | | 60 | F: AAGTAACGGAAGCCTACAGAAAC  R: ATCTTCATGCCGTCTGGTCAG |
| *MXI1* | | 60 | F: GGAAAAGAATCGACGAGCTCAT  R: GGGTGCAGTCTGGTCCTAGTG |
| *HSPA9* | | 60 | F: AGCTGGAATGGCCTTAGTCAT  R: CAGGAGTTGGTAGTACCCAAATC |
| *RHBDD2* | | 60 | F: GGTGTTTGGCATGGTTGTG  R: CGATGGAATAGCAGTAGGTGAG |
| *P53* | | 60 | F:GGCTCTGACTGTACCACCATCCA  R:GGCACAAACACGCACCTCAAAG |
| *BAX* | | 60 | F: CCTTTTCTACTTTGCCAGCAAAC  R: GAGGCCGTCCCAACCAC |
| *BCL2* | | 60 | F: ATGTGTGTGGAGAGCGTCAACC  R: TGAGCAGAGTCTTCAGAGACAGCC |
| *U6* | | 60 | F:GCTTCGGCAGCACATATACTAAAAT  R: CGCTTCACGAATTTGCGTGTCAT  RT: CGCTTCACGAATTTGCGTGTCAT |
| *GAPDH* | | 60 | F: TGGTATCGTGGAAGGACTCA  R: GGGCCATCGACAGTCTTC |

Table S5 Sequence of miR-let7a-3p, *COPB2* exon 5 and *COPB2* exon 22

| Gene | Sequence |
| --- | --- |
| miR-let7a-3p | CUAUACAAUCUACUGUCUUUC |
| COPB2 exon5 | ATGACATGCTTATTAAGCTCTGGGACTGGGATAAAAAATGGTCTTGCTCACAAGTGTTTGAAGGACACACCCATTATGTTATGCAGATTGTGATCAACCCCAAAGATAACAATCAGTTTGCCAGTGCCTCTTTGGACAGGACTATCAAG |
| COPB2 exon22 | AGTTTACTCGAACTAGAAGTAGATTTGGATAATTTGGAATTAGAAGATATTGACACAACAGATATCAATCTGGATGAAGATATTTTGGATGATTGACTGTAATGCTTTCCATTTACCTGACTAAACAGATCATTATTATATATAGGTATTGATTGCTACCCTGACCACAGTGCTTTGGACTATGAGAAACTTCTTAGATTTTTATATGTAAATGCTGTGGACCACTGGGAGCACAATGCCCACATCATCTTAAGAAGAGTTTATGTGCAGCATTTAAATCACTGTGTTTTCCTTGTTAACTAAAACAGACATGGGCTTTGATTTTTTTCATACTATTAGACCATATCTCATAAAACCTTTTGAATTAATGAAGGTACTTGTTTCCTTTCTCAATAATGAAAATAGGCTTCTAGTTTTAGAAGGCTGAGCCGAAACTACACCTTGCCTAGGGATCAGCCCCACTGTCTTTTCTTTGTATAACTAAATCTGCATTTTCAAATGTTGTCAATCACATTTTTCTTAGAGCTGAATATCCAGGCTGTAATTCTCTAAA |
